# Folic acid modifies the shape of epithelial cells during morphogenesis via a Folr1 and MLCK dependent mechanism

**DOI:** 10.1242/bio.041160

**Published:** 2019-01-15

**Authors:** Jessica B. Martin, Maria Muccioli, Kenneth Herman, Richard H. Finnell, Timothy F. Plageman

**Affiliations:** 1 The Ohio State University, College of Optometry, Columbus, OH 43210, USA; 2 Departments of Molecular and Cellular Biology and Medicine, Baylor College of Medicine, Houston, TX 77030, USA

**Keywords:** Folic acid, Myosin, Mlck, Folr1, Neural tube, Shroom3, Apical constriction

## Abstract

Folic acid supplementation can prevent neural tube defects, but the specific molecular mechanisms by which it does have not been elucidated. During neural plate morphogenesis, epithelial cell apical constriction cooperates with other events to drive tissue-bending, and when defective, can result in neural tube defects. A Rho-kinase deficient binding mutant of the apical constriction regulating protein, Shroom3 (Shroom3^R1838C^), is one of only a handful of mouse mutant lines with neural tube defects that can be rescued by folic acid supplementation. This provided a unique opportunity to probe the functional rescue of a protein linked to neural tube development by folic acid. Utilizing an epithelial cell culture model of apical constriction*,* it was observed that treatment with exogenous folic acid, as well as co-expression of the folic acid receptor *Folr1*, can rescue the function of the Rho-kinase binding deficient mutant of Shroom3 *in vitro*. It was also determined that the rescuing ability of folic acid is RhoA and Rho-kinase independent but myosin light chain kinase (MLCK) and Src-kinase dependent. Inhibition of Rho-kinase-dependent apical constriction in chick embryo neural epithelium was also observed to be rescued by exogenous folic acid and that treatment with folic acid is accompanied by elevated activated myosin light chain and MLCK. Furthermore, doubly heterozygous mouse embryos lacking one copy each of Shroom3 and Folr1 exhibit a low rate of neural tube defects and also have lower levels of activated myosin light chain and MLCK. These studies suggest a novel mechanism by which folic acid modifies epithelial cell shape during morphogenesis, shedding light onto how folic acid may prevent neural tube defects.

## Abstract (precis)

**Summary:** This manuscript describes a putative mechanism by which folic acid modifies epithelial cell shape during morphogenesis, shedding light onto the mechanism by which folic acid may prevent neural tube defects.

## INTRODUCTION

Neural tube defects (NTDs) are a group of structural birth defects characterized by a failure of the early neural plate to undergo its normal morphogenetic program leading to spina bifida, anencephaly, and craniorachischisis. Notably, maternal dietary folic acid supplementation reduces the risk of NTDs in offspring of human populations throughout the world (Group, 1991; Berry et al., 1999; López-Camelo et al., 2005; De Wals et al., 2007). In some animal models, maternal folic acid supplementation similarly decreases the frequency of NTDs (Harris, 2009; Harris and Juriloff, 2010; Marean et al., 2011). Although folic acid appears to be a powerful aid in the global effort to prevent birth defects, the specific cellular mechanisms by which folic acid prevents them remain unidentified.

In mammals, folate is transported across cell membranes through three major folate transport protein types: the reduced folate carrier, the proton-coupled folate transporter, and the folate receptors. There are three folate receptors encoded by the genes *FOLR1*, *FOLR2*, and *FOLR3* in humans and two in mice (*Folr1*/*Folr2*) that mediate folic acid intake through endocytosis (Zhao et al., 2011; Chen et al., 2013). The predominant folate receptor that is required for normal neural tube closure appears to be Folr1 as neural tube defects are observed in homozygous mouse embryos lacking this gene but not in the *Folr2* homozygous embryos (Piedrahita et al., 1999). Folr1 expression is enriched in the neural epithelium of mouse embryos and Folr1 protein (Folate receptor alpha) is localized apically during neural tube closure (Barber et al., 1999; Saitsu et al., 2003; Kur et al., 2014). In Folr1 morpholino-treated *Xenopus* embryos neural tube closure defects occur due to the failure of neural epithelial cell apical constriction, or the lack of adopting a wedge-like shape (Balashova et al., 2017). Because the cellular mechanisms that regulate apical constriction are thought to be key in the morphogenesis of the neural tube (Nikolopoulou et al., 2017), an intriguing possible mechanism for the action of folic acid could be through the regulation of apical constriction.

A major component of the apical constriction machinery in numerous vertebrate tissues is the cytoskeletal protein Shroom3, an F-actin and Rho-kinase binding protein that facilitates non-muscle myosin activation and subsequent contraction of the apical cellular junctions (Haigo et al., 2003; Nishimura and Takeichi, 2008; Chung et al., 2010; Plageman et al., 2010; Plageman et al., 2011; Ernst et al., 2012; Das et al., 2014). Shroom3 functions by recruiting Rho-kinase to apical cell junctions, facilitating the activation of non-muscle myosin II and actomyosin contraction thereby reducing the apical area of epithelial cells. Loss of function mutations in the SHROOM3 gene of humans and mice result in NTDs that include exencephaly, anencephaly, and spina bifida (Hildebrand and Soriano, 1999; Lemay et al., 2015). The importance of Rho-kinase binding to Shroom3 function is highlighted by the finding that a missense mutation of Shroom3 that inhibits Rho-kinase binding (Shroom3^R1838C^) also causes NTDs that are similar to the mouse loss of function allele (Marean, et al., 2011; Das et al., 2014; Zalewski et al., 2016). Interestingly, the phenotype in *Shroom3^R1838C/R1838C^* homozygous embryos can be partially alleviated by folic acid supplementation (Marean et al., 2011). Given this result and the knowledge of how this mutation inhibits Shroom3 function, it provides a unique opportunity to probe the mechanism of folic acid rescue of NTDs.

In this study, the mechanism of folic acid rescue of Shroom3 function was analyzed using both a cell culture model of apical constriction and mouse and chicken embryos. It was determined that folic acid and the folic acid receptor, Folr1, can rescue the function of the Rho-kinase-binding deficient mutation of Shroom3. Chemical inhibition experiments support the role for myosin light chain kinase (MLCK) mediating the functional rescue in cell culture. Further investigation demonstrated that folic acid can also rescue non-muscle myosin activation and apical constriction in embryos treated with a Rho-kinase inhibitor. The effect was also coincident with an increase in junctional MLCK activation in response to folic acid. Finally, it was determined that both non-muscle myosin and MLCK activation are decreased in Shroom3/Folr1 doubly heterozygous embryos. These results provide details of a potential mechanism by which folic acid facilitates morphogenesis and/or prevents disruptions in this process in developmental defects.

## RESULTS

### Exogenous folic acid and Folr1 expression rescues the function of the Rho-kinase binding mutation of Shroom3

**Fig. 1.**
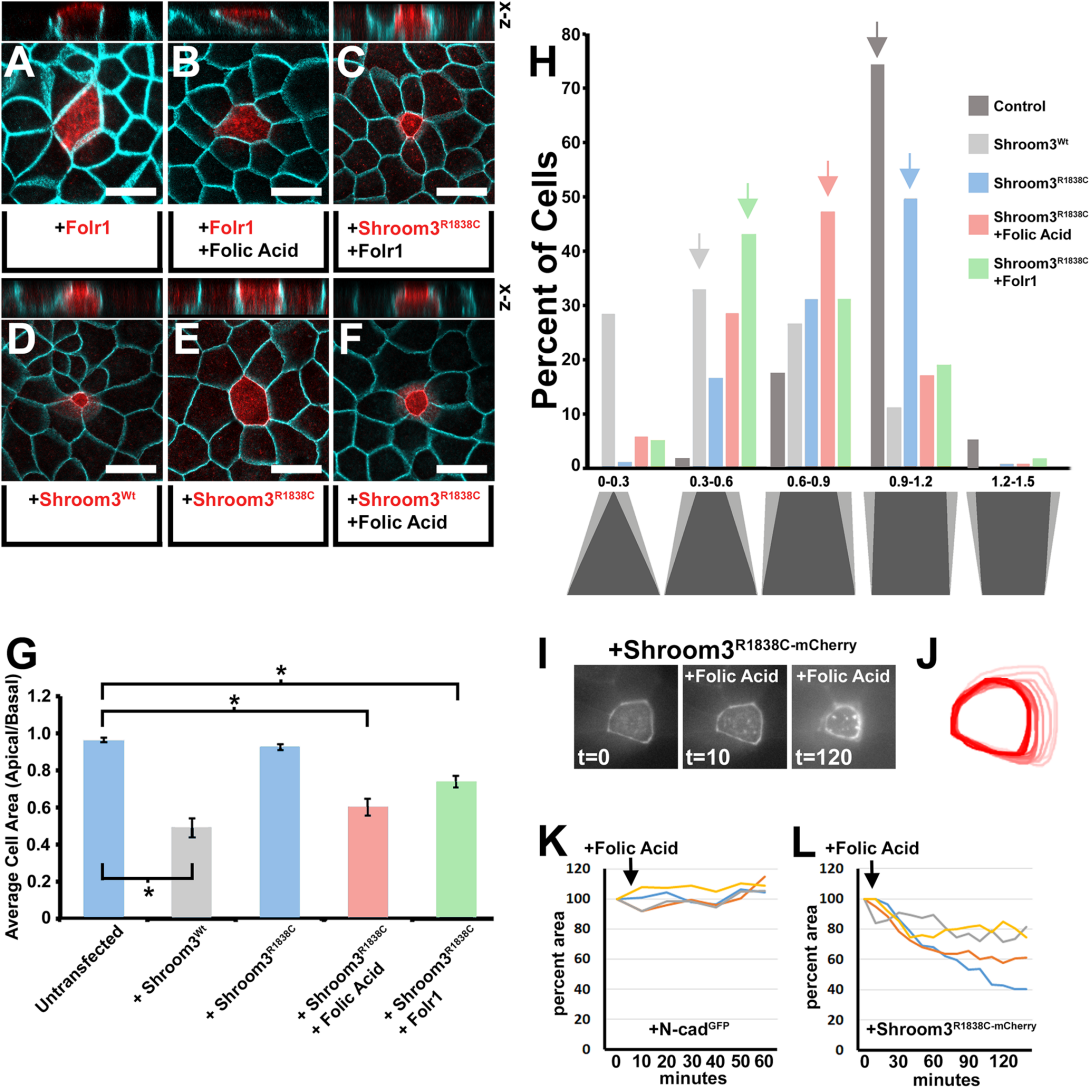
**Exogenous folic acid and Folr1 rescues the** **function of a Rho-kinase binding mutation in Shroom3.** (A–F) Apical views of MDCK cells transfected with the indicated expression vector, incubated with or without exogenous folic acid (100 µM) and immunofluorescently labeled with β-catenin (turquoise) and either Shroom3 or Folr1 antibodies (red). Above each panel is a virtual section through the transgenic cell in the x-z plane. Scale bar: 20 µm. (G) The mean apical basal area ratio (ABAR) was calculated from area measurements of apical and basal images of transgenic cells and depicted in the graph. Asterisks indicate data sets with significantly reduced ABAR ratios *P*<0.01. (H) The shapes depict the approximate shape range of cells with the indicated ABARs in increments of 0.3. The percentage of cells within specific ABAR increments are represented for the indicated experimental groups. Arrows mark the ABAR increment group with the greatest number of cells. Note that the experimental groups have distinct peak ABAR increment ranges indicating a shift in the population. (I) Time-lapse images of a Shroom3^R1838C^-mCherry tagged transgenic cell before and after the addition of folic acid (100 µm). (J) The apical outline of the transgenic cell in I was traced at each time point and superimposed to show the radial reduction of apical area. (K–L) The change in apical area was plotted with time following folic acid treatment of four representative transgenic cells expressing either N-cadherin-GFP or Shroom3^R1838C^-mCherry.

To examine the relationship between folic acid internalization and epithelial cell shape, the MDCK (Madin-Darby Canine Kidney) cell culture model of apical constriction (AC) was utilized (Haigo et al., 2003). While previous studies have demonstrated that the folic acid receptor, Folr1, is required for apical constriction in the neural plate of *Xenopus* embryos (Balashova et al., 2017), Folr1 expressed in MDCK cells is not sufficient to induce AC in the presence or absence of exogenously added folic acid (Fig. 1A–B, Table 1) as determined by calculating the mean ratio of the apical and basal areas of transgenic cells. Although the Folr1 protein (Folate receptor alpha) localizes to the apical membrane and is in position to possibly affect the AC of MDCK cells (Fig. 1A–B), the apical/basal area ratio (hereafter ABAR) of Folr1 positive cells remained close to 1, similar to that of untreated cells (Table 1).

**Table 1.**
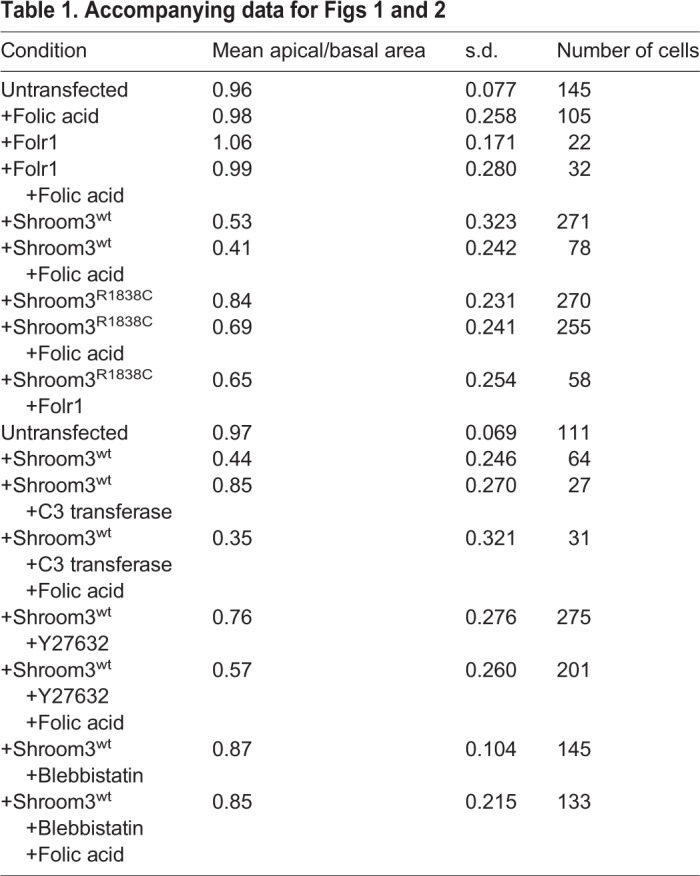
**Accompanying data for Figs 1 and 2**

**Fig. 2.**
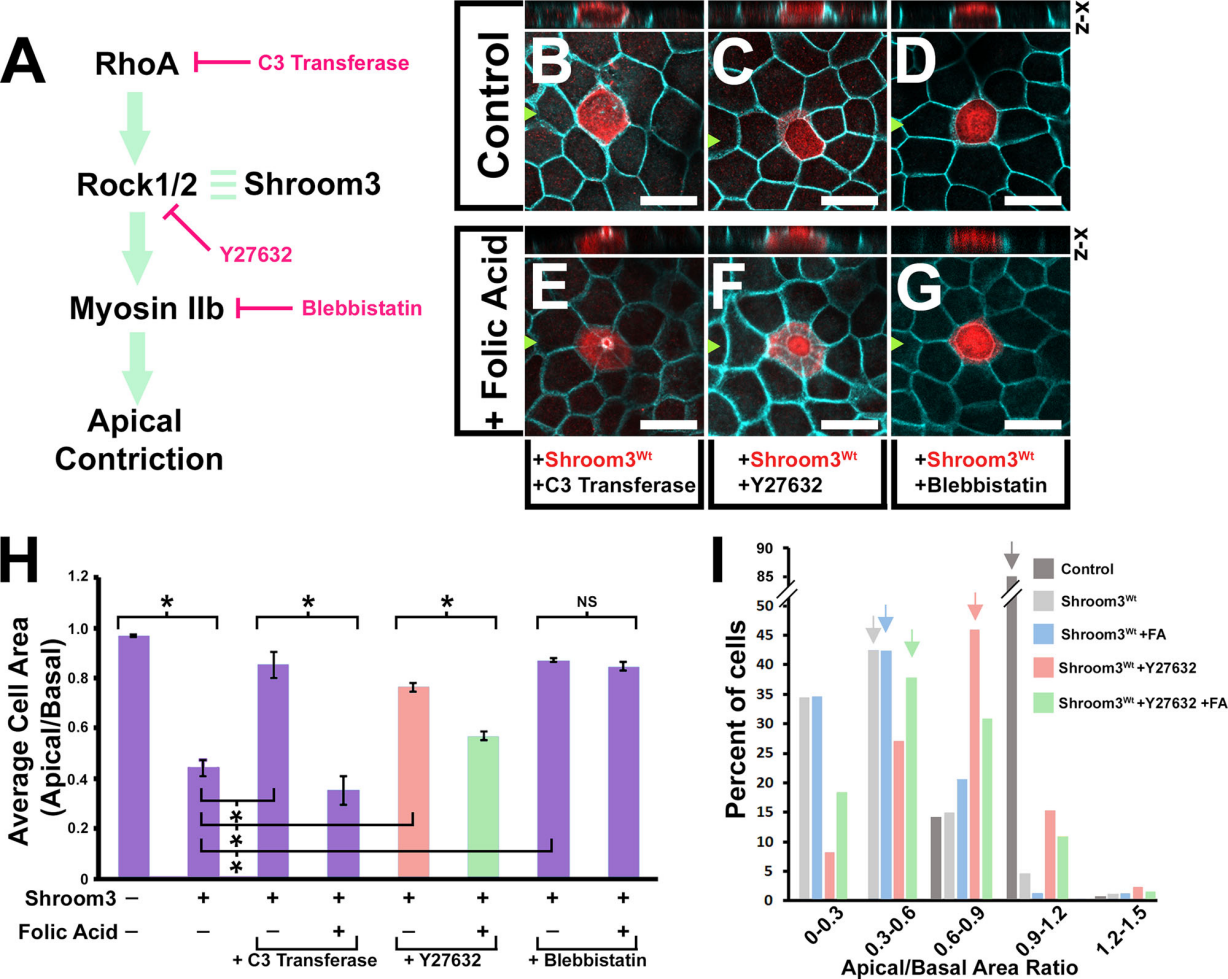
**Folic acid rescue is independent of RhoA and Rho-kinase activity.** (A) A diagram of the Shroom3-dependent apical constriction pathway marked with the locations of chemical inhibition. (B–G) Apical views of MDCK cells transfected with a Shroom3^wt^-expression vector, incubated with or without exogenous folic acid (100 µM) and the indicated chemical inhibitor, and immunofluorescently labeled with β-catenin (turquoise) and Shroom3 antibodies (red). Above each panel is a virtual section through the transgenic cell in the x-z plane. Scale bars: 20 µm. (H) The mean apical basal area ratio (ABAR) was calculated from area measurements of apical and basal images of transgenic cells and depicted in the graph. Asterisks indicate data sets with significant changes in ABAR ratios *P*<0.01. (I) The percentage of cells within specific ABAR increments are represented for the indicated experimental groups. Arrows mark the ABAR increment group with the greatest number of cells. Note that the experimental groups have distinct peak ABAR increment ranges indicating a shift in the population.

Because folic acid is capable of rescuing the neural tube defect phenotype in a percentage of Rho-kinase-binding deficient Shroom3 mouse mutant (*Shroom3^R1838C/R1838C^*) embryos (Marean et al., 2011; Das et al., 2014), MDCK cells were again used to test the role of Folr1 and folic acid on Shroom3-dependent AC. When exogenous folic acid is added to the media of Shroom3^wt^+ MDCK cells the mean ABAR decreases (0.53 compared with 0.41), suggesting that folic acid can have an additive effect on apical constriction (Table 1). However the population distributions of the data from the Shroom3^wt^+ and Shroom3^wt^+/folic acid experimental groups did not change much (Fig. 2H). As previously shown, Shroom3-induced AC is attenuated by presence of the R1838C mutation and the mean ABAR increases from 0.53 to 0.84 (Fig. 1D–E,G, Table 1) (Das et al., 2014). In the presence of folic acid or the co-expression of transgenic Folr1, AC is rescued in MDCK cells that express Shroom3^R1838C^ (Fig. 1C,F,G; Fig. S1A–B) and the mean ABAR is significantly decreased relative to non-transfected cells (0.69 and 0.65, respectively) (Fig. 1G, Table 1). Because individual cell shapes in the MDCK AC assay can vary, and that greater transgenic junctional Shroom3 intensity correlates with decreases in ABAR (Fig. S2A), the percent of transgenic cells with specific ABARs were also determined and plotted onto a distribution graph (Fig. 1H). Although on average more junctional Shroom3 is observed in cells that have undergone greater AC, live imaging and quantification of junctional intensity during Shroom3-induced MDCK apical constriction suggests that it is likely due to increasing the local protein concentration along the junction rather than protein expression-dependent effects (Fig. S2B–D). As expected, the ABAR range increment with the largest percentage of untransfected cells is 0.9–1.2 in contrast to those transfected with Shroom3^wt^, which was 0.3–0.6, indicating that most of the cells have markedly apically constricted (Fig. 1H, grey arrows). Most Shroom3^R1838C^ transfected cells fell into the range of 0.9–1.2 indicating that most cells did not undergo substantial AC (Fig. 1H, blue arrow). However, in the presence of folic acid or co-transfected Folr1, the population shifted into the 0.6–0.9 and 0.3–0.6 ranges, respectively (Fig. 1H, green and red arrows) indicating that most cells in these experimental groups underwent AC. To further verify that folic acid is capable of rescuing the ability of Shroom^R1838C^ to induce AC, live fluorescent microscopy was performed on transgenic cells in the presence of folic acid (Fig. 1I–L; Movie 1). Upon dosing the cell culture media with exogenous folic acid, Shroom^R1838C^ transgenic cells but not control cells progressively decreased their apical area. Together, these data suggest that folic acid via Folr1 function can bypass the Rho-kinase binding deficiency of Shroom3^R1838C^. In addition, these results are consistent with the ability of folic acid to partially rescue the *Shroom3^R1838C/R1838C^* mutant embryonic phenotype in mice and suggest rescue may be due to an influence on apical constriction (Marean et al., 2011).

### Folic acid-mediated rescue is at least partially independent of RhoA and Rho-kinase

To ascertain the mechanism of folic acid rescue of Shroom3^R1838C^ functional deficiency, chemical inhibitors of known canonical AC pathway components targeting RhoA (C3 Transferase), Rho-kinase (Y-27632), and non-muscle myosin II (Blebbistatin) were utilized (Fig. 2A). MDCK cells were transiently transfected with Shroom3^wt^ and the media dosed with the inhibitors with and without exogenous folic acid (100 µM). As expected, each of the inhibitors attenuated Shroom3-dependent AC (Fig. 2B–D), and the mean ABARs were significantly increased (Fig. 2H, Table 1). In the presence of folic acid and either C3 transferase or Y-27632, AC was restored as assessed by the mean ABARs (0.35 and 0.56, respectively). The ability of folic acid to rescue Y-27632 is further illustrated by examining the population of cells which revealed that most Shroom3^wt^-positive cells treated with Y27632 had an ABAR of 0.6–0.9 (Fig. 2I, red arrow), while the addition of folic acid caused most cells to have a 0.3–0.6 ABAR (Fig. 2I, green arrow). However, blebbistatin inhibition could not be restored by exogenous folic acid (Fig. 2G,H, Table 1) suggesting that folic acid cannot bypass the requirement for myosin IIb activity during Shroom3-dependent AC. Importantly, these results demonstrate that folic acid integrates itself within the AC pathway upstream of myosin II activity and is at least partially independent of RhoA or Rho-kinase activity.

### Folic acid alleviates the apical constriction deficiency in the chick neural plate following rho-kinase inhibition

**Fig. 3.**
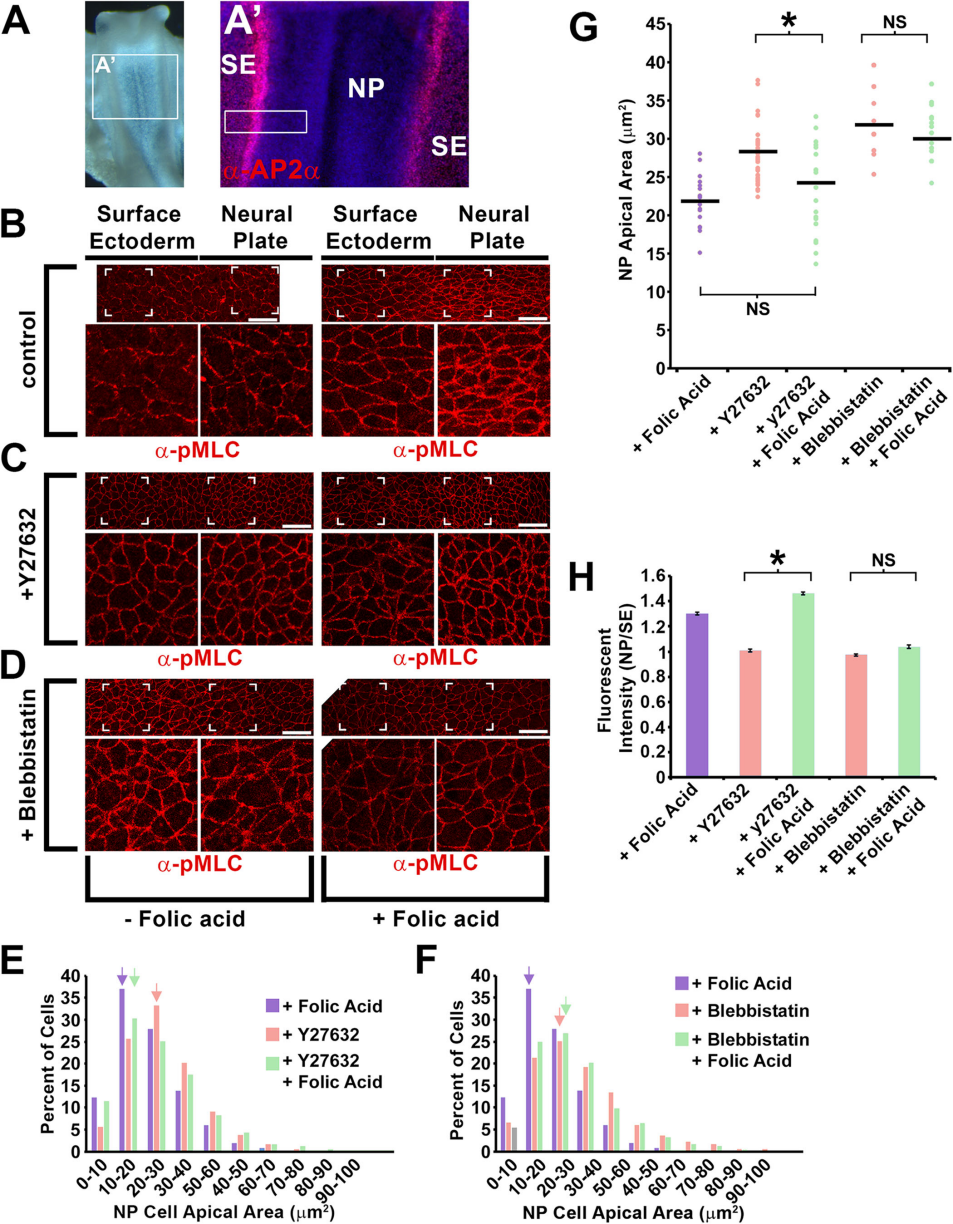
**Exogenous folic acid rescues neural epithelial apical constriction and activation of non-muscle myosin upon Rho-kinase inhibition.** (A) Stage 7 chick embryo indicating the region of analysis. The magnified image to the right is immunofluorescently labeled with an antibody specific to the surface ectoderm (AP2α, red) to differentiate them from neural epithelial cells. The white box indicates an approximate region analyzed in B–D. (B–D) Stage 7 chick embryos incubated for 2 h *ex vivo*, with or without exogenous folic acid (100 µm) and with or without Y27632 or blebbistatin were fixed and immunofluorescently labeled with an antibody specific for the phosphorylated/active form of myosin light chain (pMLC). The images show a region near the surface ectoderm/neural epithelial border and the square hatches indicate the area magnified below each panel. Scale bars: 25 µm. (E–F) All of the neural epithelial cell apical areas of embryos from each experimental group were measured and the percent of cells with areas in 10 µm^2^ increment ranges was tabulated and depicted in the graph. Note that the peak population of neural epithelial cells treated with Y-27632 and folic acid shifts toward a smaller range increment, while this does not occur in the blebbistatin and folic acid treated group (arrows). (G) The distribution of means from all individual regions of all embryos analyzed within each experimental group are depicted. The mean value is depicted by the horizontal line. (H) The fluorescent intensity of epithelial cell bi-cellular junctions in the neural epithelial and surface ectoderm were measured and the mean ratio of intensities of the neural epithelial and surface ectoderm from each experimental group are depicted. The asterisks indicate data sets with significant differences (*P*<0.01); NS, not significant.

Inhibition of Rho-kinase activity with Y-27632 disrupts neural tube closure in chick and mouse embryo culture and causes neural epithelial cells to increase their apical areas (Wei et al., 2001; Nishimura and Takeichi, 2008; Gray and Ross, 2011). To determine if folic acid can similarly rescue AC following Y-27632 treatment *in vivo*, stage 7 chick embryos were incubated for 90 min in Y-27632 or blebbistatin in the presence or absence of exogenous folic acid. Embryos were subjected to wholemount immunolabeling with an antibody specific to AP2α, a surface ectodermal marker (Fig. 3A) and the phosphorylated, active form of the myosin II light chain (pMLC) which can label the apical junctions of neural plate and surface ectodermal cells (Fig. 3B–D). Upon measuring the apical areas of neural epithelial cells using β-catenin immunolabeling as a guideline, the addition of Y-27632 caused neural epithelial cell apical areas to increase compared with cells from control embryos with more cells having an apical area in the range of 20–30 µm^2^ versus a range of 10–20 µm^2^ among all cells measured (Fig. 3E, blue versus red arrows), indicating that the contractility of the apical junctions relaxed. With the addition of Y-27632 and folic acid, most cells were found to have an apical area in the 10–20 µm^2^ range, similar to the control group (Fig. 3E, green arrow). When the same experiment was performed with blebbistatin (Fig. 3D), folic acid could not rescue neural epithelium apical area and the peak apical area range increment was 20–30 µm^2^ with or without folic acid (Fig. 3F, red versus green arrows). A similar result is observed when comparing the distribution of cell area means from individual regions of several embryos (Fig. 3G). These data suggest that similar to MDCK cells, folic acid can rescue AC in the neural epithelium of chick embryos when Rho-kinase activity but not myosin II activity is inhibited. The intensity of junctional pMLC was also compared between experimental groups as a marker for myosin II activation. Immunolabeling of pMLC is normally greater in the neural epithelium cell apical junctions versus the surface ectoderm but the addition of Rho-kinase or myosin II inhibitors diminished the neural epithelial cell apical junctional intensity (Fig. 3C–D,H). Similar to the effect on AC, pMLC junctional intensity was increased in the junctions of neural epithelial cells treated with folic acid and Y-27632 but not with folic acid and blebbistatin. This result suggests that folic acid can induce AC through the activation myosin II activity at least partially independent of Rho-kinase *in vivo*.

### Folic acid mediated rescue is dependent on myosin light chain kinase *in vitro* and *in vivo*

**Fig. 4.**
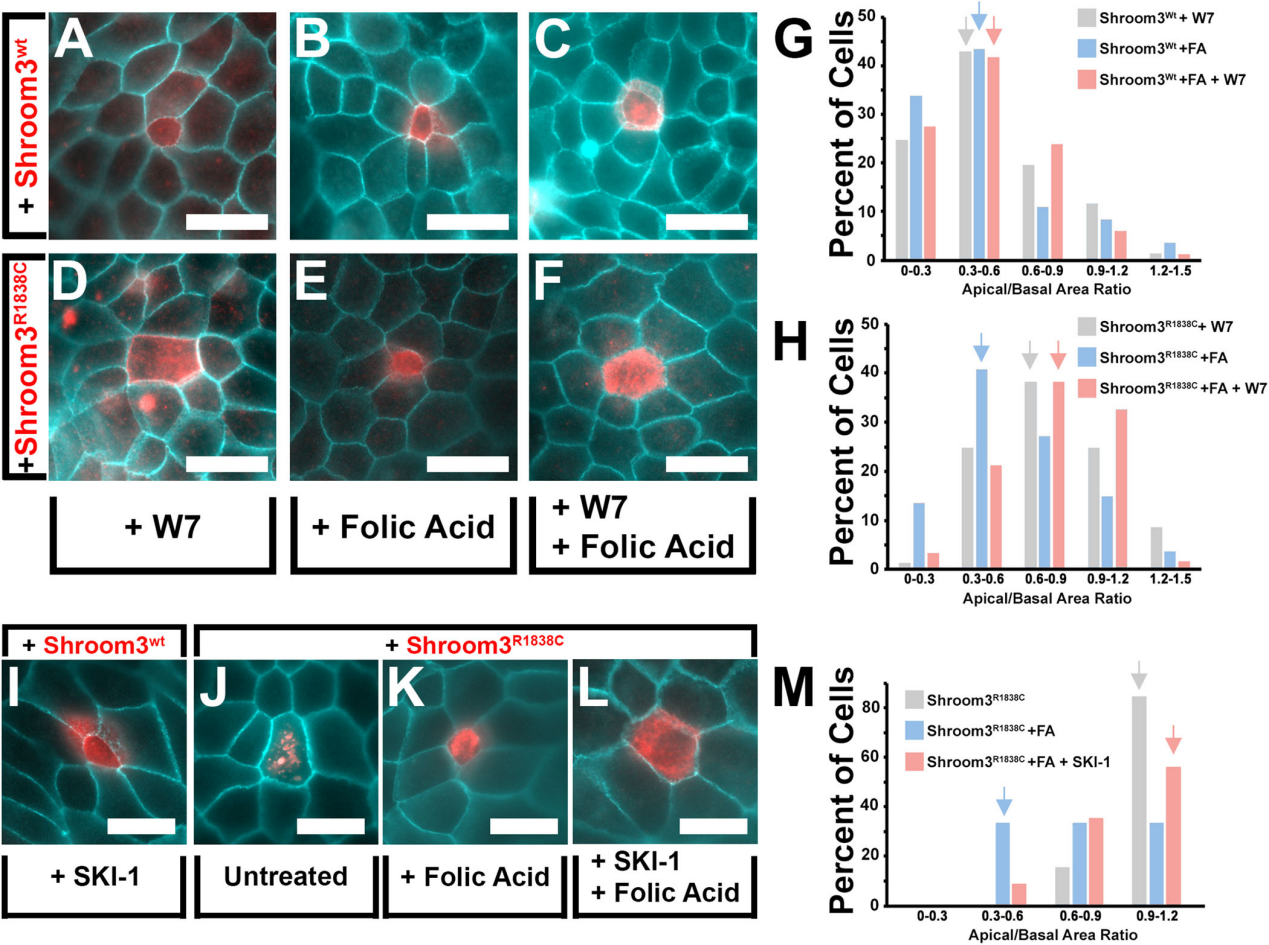
**Folic acid rescue is MLCK and Src-kinase dependent.** (A–F) Shroom3^wt^ or Shroom3^R1838C^ transgenic MDCK cells were treated for 2 h with or without exogenous folic acid (100 µm), and with or without an MLCK inhibitor (W7). Cells are immunofluorescently labeled with β-catenin (turquoise) and Shroom3 antibodies (red). (G–H) The ABAR was calculated for the indicated experimental groups and depicted on each graph. Note that W7 does not affect Shroom3^wt^ (G), but does inhibit the ability of folic acid to rescue the ABAR of Shroom3^R1838C^ positive cells (H). (I–L) Shroom3^wt^ or Shroom3^R1838C^ transgenic MDCK cells were treated for 2 h with or without exogenous folic acid (100 µm), and with or without a Src inhibitor (SKI-1). Cells are immunofluorescently labeled with β-catenin (turquoise) and Shroom3 antibodies (red). (M) The ABAR of was calculated for the indicated experimental groups and depicted on the graph. Note that SKI-1 inhibits folic acid rescue of Shroom3^R1838C^ positive cells. Scale bars: 20 µm.

Because it was observed that pMLC junctional localization increases with the addition of folic acid, it was hypothesized that folic acid may activate a distinct pathway that is capable of phosphorylating myosin II. Myosin light chain kinase (MLCK) phosphorylates myosin light chain at the same amino acids as Rho-kinase (Ikebe and Hartshorne, 1985; Amano et al., 1996) and has been implicated in regulating AC (Lee and Harland, 2007; Homem and Peifer, 2008; Blanchard et al., 2010). To determine if the folic acid mediated effect is MLCK dependent, an inhibitor of Calmodulin and MLCK activity, W7, was used to see what affect it had on Shroom3-dependent AC. W7 was not observed to inhibit Shroom3-dependent AC in MDCK cells (Fig. 4A–C,G, Table 2), similar to previous studies (Hildebrand, 2005). However, it was observed that W7 does attenuate the ability of folic acid to rescue the induction of AC by the Shroom3 Rho-kinase binding mutant (Fig. 4E versus 4F, 4H, Table 2). While most cells from the folic acid rescued experimental group have an ABAR in the range of 0.3–0.6, most of the Shroom3^R1838C^ positive cells co-incubated with folic acid and W7 have an ABAR in the 0.6–0.9 range (Fig. 4H). Similar results were obtained using the alternative MLCK inhibitor ML-7 (Table 2; Fig. S3). In both cases, statistically significant differences between the experimental groups comprising the folic acid treated Shroom3^R1838C^ cells with or without W7 and ML-7 drug treatment were observed. These data suggest that the mechanism of folic acid rescue of AC is dependent on MLCK activity. Because Src-kinase functions with MLCK in several contexts (Webb et al., 2004; Barfod et al., 2011; Samak et al., 2014) its role in folic acid mediated rescue of Shroom3 function was assessed. Like W7, the Src inhibitor SKI-1 does not inhibit Shroom3^wt^-induced AC, but does attenuate the ability of folic acid to rescue Shroom3^R1838C^ mediated AC (Fig. 4I–M, Table 2) indicating that Src activity is necessary for the effect mediated by folic acid.

**Table 2.**
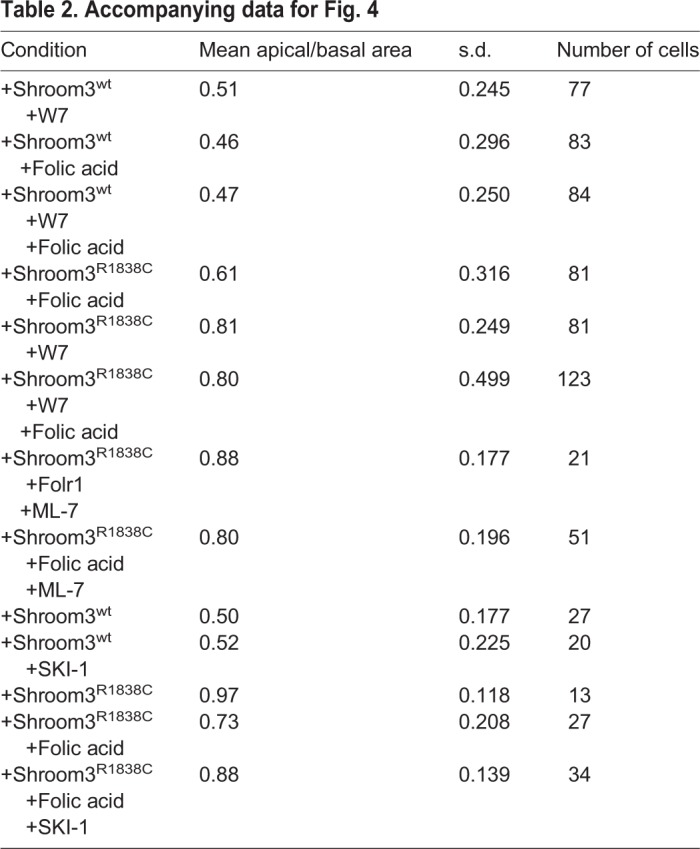
**Accompanying data for Fig. 4**

**Fig. 5.**
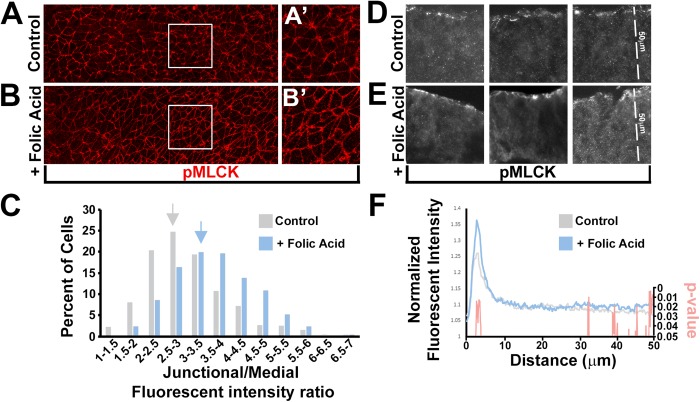
**Folic acid increases apical junctional pMLCK localization *in vivo*.** (A–B) Stage 7 chick embryos incubated for 2 h *ex vivo* with or without exogenous folic acid (100 µm) and immunofluorescently labeled with pMLCK antibody (red). (C) Junctional and medial fluorescent intensity was measured from neural epithelial images and the junctional/medial intensity ratios were calculated. A population graph depicting the percentage of cells with specific increments of intensity ratios demonstrates that the folic acid experimental group has the most cells in an increment range that is greater than the control group (arrows). (D–E) Representative images of cryosections through the neural epithelial of embryos incubated *ex vivo* with or without folic acid for 2 h and immunofluorescently labeled with a pMLCK antibody. (F) The fluorescent intensity along a 50 µm apical/basal line was measured from several images of cryosections similar to D–E and the mean intensity along the line was calculated. Control and folic acid treated embryos were compared and Student's *t*-test was performed between groups at each position along the 50 µm line. The *P*-value at each position was plotted simultaneously with the normalized intensity value to demonstrate regions with significant differences. Note that the intensities differ near the apical junctional complex.

To determine if MLCK acts downstream of folic acid *in vivo*, stage 7 chicken embryos were incubated with or without folic acid for 2 h and immunolabeled with an antibody specific for the activated, phosphorylated form of MLCK (pMLCK) (Fig. 5A–B). Folic acid treated embryos were observed to have greater levels of apical junctional signal when assessed by immunolabeling whole embryos (Fig. 5A–C), or cryosections through the neural epithelium (Fig. 5F). To quantify this difference, several sections from multiple embryos were immunolabeled with pMLCK and β-catenin antibodies and the average intensity along a 50-µm-long apical-basal line was then determined. The normalized peak fluorescent intensity of pMLCK but not β-catenin was significantly enhanced in sections from the folic acid treated group (*P*<0.05) suggesting that folic acid can stimulate the recruitment and or activation of MLCK to the apical junctional complex (Fig. 5F; Fig. S4).

### Shroom3 and Folr1 function together to promote non-muscle myosin and MLCK activation and neural tube closure

**Fig. 6.**
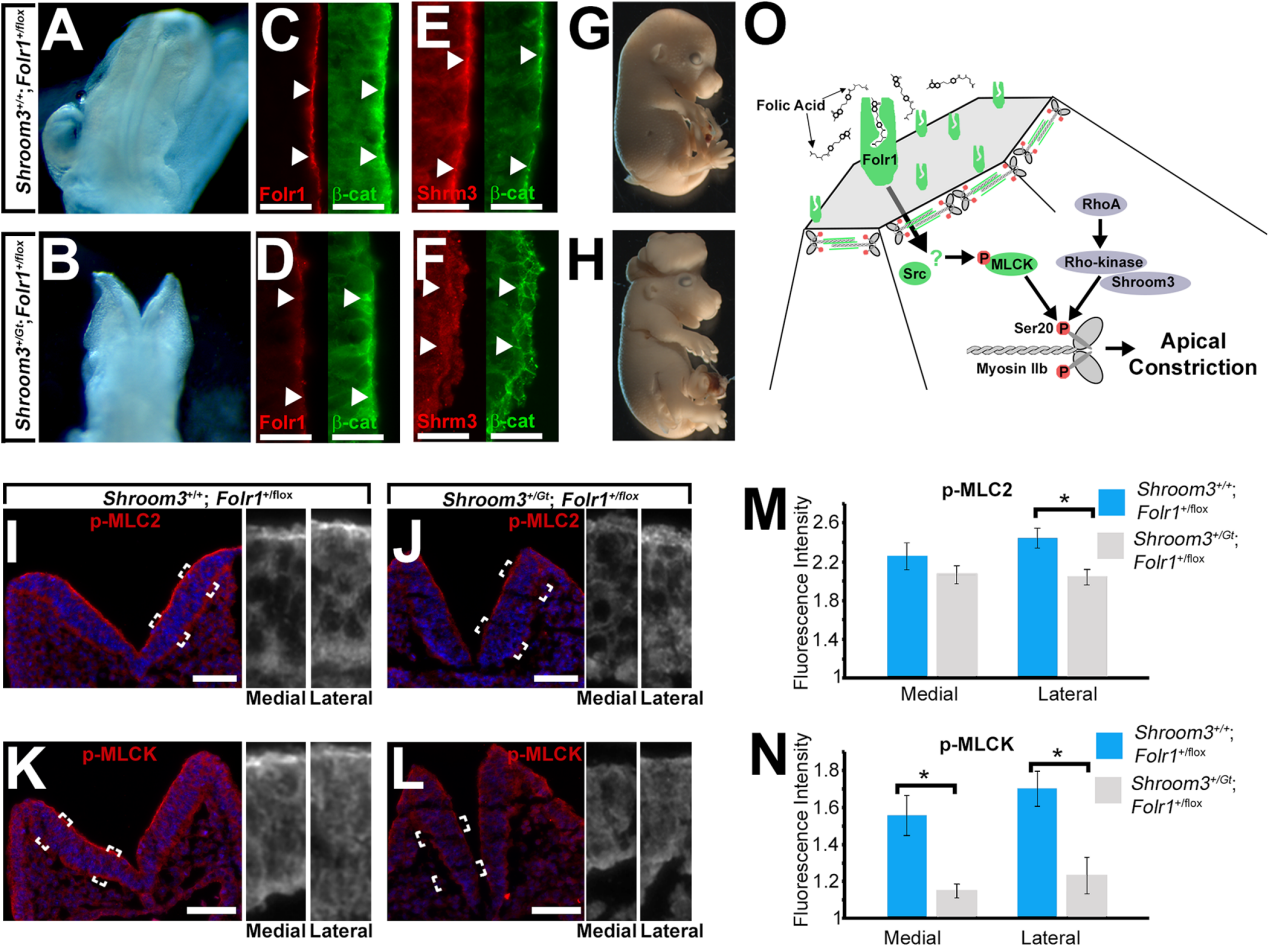
**Apical junction pMLC and pMLCK and neural tube closure are dependent on combined Shroom3 and Folr1 function.** (A–B) E9.5 embryos of control and doubly heterozygous embryos (deficient of 1 allele of Shroom3 and Folr1 genes). (C–F) Cryosections through the neural epithelial of E9.5 control and double heterozygote embryos immunofluorescently labeled with antibodies specific for Folr1 and β-catenin or Shroom3 and β-catenin. The arrows mark regions of positive apical β-catenin labeling and show reduction of apical Shroom3 and Folr1 in double heterozygotes. (G–H) E15.5 embryos of control and doubly heterozygous embryos. Note the exencephaly present in the double heterozygotes. (I–L) Cryosections through the neural epithelial of E9.0 control and double heterozygote embryos immunofluorescently labeled with antibodies specific for phosphorylated myosin light chain (pMLC) or phosphorylated myosin light chain kinase (pMLCK) (red) and Hoechst (blue). The hatched boxes indicate regions magnified in the panels to the right. (M–N) The mean apical fluorescent intensity of pMLC and pMLCK was calculated from measurements of several cryosections and depicted in the graph. Asterisks indicate experimental groups with significantly reduced intensities (*P*<0.01). (O) A model of a proposed pathway by which folic acid exerts its effect on Shroom3-dependent apical constriction.

To further confirm the relationship between Folr1 and Shroom3 function during neural epithelial morphogenesis, doubly heterozygous mouse embryos (*Shroom3^+/Gt^; Folr1^+/flox^*) were generated and analyzed. The *Shroom3^Gt^* allele expresses cre from the endogenous *Shroom3* promoter, and is capable of mediating recombination in Shroom3-expressing tissues when in the presence of a floxed allele (Hildebrand and Soriano, 1999; Lang et al., 2014). Neither Shroom3 nor Folr1 germline heterozygote embryos are reported to have NTDs, however a relatively small rate of NTDs (4/42) were observed in *Shroom3^+/Gt^; Folr1^+/flox^* embryos (Fig. 6A–H). The intensity of Shroom3 and Folr1 protein is reduced at the apical side of the neural epithelial tissue in E9.5 *Shroom3^+/Gt^; Folr1^+/flox^* (Fig. 6C–F) embryos. To determine if the absence of some of the Shroom3 and Folr1 protein may have an effect on the phosphorylation and activation of myosin II and/or MLCK, neural epithelial sections of control and E9.0 *Shroom3^+/Gt^; Folr1^+/flox^* embryos were immunolabeled with antibodies specific for pMLC and pMLCK (Fig. 6I–L). In the lateral neural epithelial, pMLC and pMLCK immunolabeling was less intense in *Shroom3^+/Gt^; Folr1^+/flox^* embryos compared with control embryos (Fig. 6N–O). These data suggest that Shroom3 and Folr1 may function together to ensure sufficient activation of myosin II in part through the activation of MLCK.

## DISCUSSION

### Overview of results

Collectively these data support a role for folic acid to facilitate apical constriction and posit a potential mechanism for folic acid prevention of neural tube defects that is diagrammed in Fig. 6O. In epithelial cells of the neural epithelium, Folr1 is thought to mediate the uptake of folic acid and trigger the activation and phosphorylation of myosin light chain kinase (MLCK) possibly through the function of Src. The activation of MLCK is then thought to phosphorylate and activate apically positioned actomyosin filaments within the apical junctional complex and facilitate apical constriction and neural epithelial morphogenesis. Furthermore, these data are consistent with the possibility that folic acid protects embryos from neural tube defects that have a deficiency of actomyosin activation within neural epithelial cells.

### A role of folic acid during apical constriction

The mechanism(s) by which folic acid helps to prevent NTDs has been an outstanding question for decades (Wallingford et al., 2013). This is perhaps due to the paucity of known animal models with NTDs that are phenotypically rescued by folic acid (Harris and Juriloff, 2010; Marean et al., 2011). Because folic acid can rescue the phenotype of *Shroom^R1838/R1838^* embryos it provided a unique opportunity to probe this mechanism (Marean et al., 2011). This mutation inhibits Shroom3's ability to induce AC and bind Rho-kinase and causes mouse embryos to exhibit NTDs (Das et al., 2014; Zalewski et al., 2016). For this reason, it was hypothesized that folic acid may function independently of Rho-kinase activity or may somehow rescue the ability of Shroom3^R1838C^ to bind Rho-kinase. While binding assays were not performed, it is unlikely that the latter is true because inhibition of Rho-kinase can be rescued by folic acid. If it was observed that folic acid rescued Shroom3 function after inhibiting RhoA but not Rho-kinase activity the latter possibility would have been more viable. It is important to point out that although Rho-kinase inhibition was rescued by folic acid the degree of rescue was not 100% leaving open the possibility that some of the effect of folic acid could be Rho-kinase dependent. This possibility is supported by the observation that folic acid can promote cell migration through the activation of RhoA (Oleinik et al., 2014).

It was also observed that neither folic acid nor the expression of the Folr1 receptor could trigger AC alone indicating that folic acid is likely not a major organizer of this process. Rather, folic acid may facilitate the rescue of the Shroom3 mutant only because the rest of the AC machinery is already in place and act like a trigger for the process. Data demonstrating that maternal dietary supplementation of folic acid has only decreased and not eliminated NTDs and that there are several mouse knockouts unprotected by supplemental folic acid are both consistent with this observation (Harris and Juriloff, 2010; Wallingford et al., 2013). Furthermore, the Shroom3-dependent accumulation of actomyosin may be wholly or partially independent of the Rho-kinase binding function of Shroom3 because the location of the acting binding domain and its actin bundling ability are in a region distinct from the Rho-kinase binding region (Hildebrand and Soriano, 1999; Hildebrand, 2005; Dietz et al., 2006; Bolinger et al., 2010).

### Folr1 function during apical constriction rescue

While transgenic Folr1 expression was not sufficient to induce apical constriction in MDCK cells, it was capable of rescuing the ability of the mutant Shroom3^R1838C^ protein to induce AC. Presumably, this is due to granting MDCK cells with an enhanced ability to internalize folic acid. The details of exactly how folr1-dependent folic acid uptake may influence the activity of cytoplasmic proteins is still unclear but endogenous folic acid receptors are found in both MDCK cells (Fig. S1C) and the mouse neural epithelium during development (Saitsu et al., 2003; Kur et al., 2014). This mechanism could involve the function of the low density lipoprotein-related protein 2 (Lrp2/megalin). Lrp2 can mediate both folr1 and folic acid uptake in the neural epithelial during neural tube closure which is thought to lead to the NTDs that occur in a proportion of Lrp2 deficient embryos (Kur et al., 2014). It is possible that the folic acid rescue mechanism we observe in this study is wholly or partially dependent on Lrp2 function. Once endocytosed, folic acid receptor proteins when bound folic acid are internalized by endocytosis, and can move into the cytosol via the protein coupled folate transporter (PCFT) (Zhao et al., 2009). A future challenge would be to define how endocytosed folr1 and folic acid can influence MLCK activity.

The preferential localization of Folr1 in the apical compartment of neural epithelial cells observed here and elsewhere is consistent with a role for this protein during AC (Kur et al., 2014; Balashova et al., 2017). While it was observed that Folr1 protein is not junctionally restricted but rather associated with the apical membrane, it is known that junctional localization is not a requirement for proteins to facilitate apical constriction. For example, when Shroom3 protein is localized specifically to the apical membrane it is capable of inducing AC *in vivo* and *in vitro* (Hildebrand, 2005; Bolinger et al., 2010). Apical cortex localized actomyosin networks have also been observed to induce AC in the neural epithelial of vertebrates and in epithelial cells participating in *Drosophila* embryo morphogenesis (Martin and Goldstein, 2014; Christodoulou and Skourides, 2015). Because Folr1 is localized to the apical membrane yet is required for neural epithelial cell AC of *Xenopus* embryos during neural tube closure, it is unlikely that junctional localization is a requirement (Balashova et al., 2017).

### MLCK regulation of actomyosin contraction

The role of MLCK was investigated primarily because it is capable of activating and phosphorylating the same amino acids (Thr18/Ser19) within the myosin light chain of non-muscle myosin as Rho-kinase (Ikebe and Hartshorne, 1985; Amano et al., 1996). However, additional kinases can also phosphorylate and activate these amino acids and their contribution cannot be ruled out (Vicente-Manzanares et al., 2009). For example, MRCK, a kinase capable phosphorylating Thr18/Ser19 of the myosin light chain, was recently reported to drive AC in *Caenorhabditis elegans* embryos making it a possible target for folic acid activation (Marston et al., 2016). Furthermore, because W7 is a calmodulin inhibitor, additional calmodulin targets not tested in this study could be involved. However, equivalent results were obtained with W7 or ML-7 inhibitor, which is specific for the inhibition of MLCK and does not affect calmodulin or other kinases. Therefore it is concluded that the action of folic acid is at least partially MLCK dependent. A role for MLCK in AC also has some precedence. Bottle cell formation in *Xenopus* embryos, which utilize AC, is MLCK dependent (Lee and Harland, 2007). Constitutively active MLCK can also induce AC in *Drosophila* epithelial cells (Homem and Peifer, 2008; Blanchard et al., 2010).

Another question that arises from this study is how folic acid activates MLCK activity. The data presented here suggest this may be through a Src dependent process. The Src family kinases are a large family of non-receptor tyrosine kinases that have been associated with apical junction actomyosin contractility (Andreeva et al., 2014; Williams et al., 2014) and can phosphorylate and activate MLCK (Birukov et al., 2001). In addition, folic acid and folr1 have been reported to activate the Src signaling pathway to mediate other cellular processes such as proliferation and migration (Lin et al., 2012; Hou et al., 2013; Kuo et al., 2015). While it is yet unclear how folic acid may affect Src, the regulation of this pathway is clearly important as genetically ablating a negative regulator of Src activity (Csk) leads to NTDs in mice (Imamoto and Soriano, 1993). Elucidating the details of the mechanism underlying folic acid regulation of MLCK activity will likely require further investigation into the regulation of Src.

Although not addressed in this study, it remains possible that folic acid and folr1 may affect additional biological pathways to protect against NTDs. As an essential molecule needed for the production of purines and thymidylate, folic acid deficiency could lead to the disruption of other crucial cellular processes necessary for development (Baggott and Tamura, 2015). However, the observations presented here suggest that the mechanism underlying the protection of at least some causes of NTDs occur through the activation of the pathways that regulate apical constriction.

## MATERIALS AND METHODS

### Transient transfection, chemical inhibitor usage and expression vector cloning

Madin-Darby Canine Kidney epithelial cells (graciously donated by Dr Andrew S. Bowman, Ohio State University) were cultured in Modified Eagle's Media, 1x Penicillin/Streptomycin, 1% non-essential amino acids (Corning; Corning, USA) and 10% Fetal Bovine Serum (Thermo Fisher Scientific). Cells were grown on Transwell Permeable Membrane Supports (Corning) at 37°C, 5% CO_2_ to approximately 50% confluence and transiently transfected with 0.1–0.5 mg plasmid DNA using TransIT-293 transfection reagent (Mirus) in accordance with the manufacturer's instructions. The Shroom3^R1838C^ (CGC-TGC) mutation was generated on the PCS2-Shroom3-Flag vector (Plageman et al., 2010) using the Quik change mutagenesis kit (Agilent Technologies). To generate the *Folr1* plasmid, PCR was utilized to amplify the coding sequence of mouse Folr1 (5′-ACCATGGCTCACCTGATGACT, 5′-TCAGCTGATCACCCAGAGCAG) from cDNA generated from RNA collected from E10.5 whole mouse embryo heads (Superscript III, Invitrogen) and the subsequent DNA fragment was cloned into the pcDNA3.1/V5-His TOPO vector (Invitrogen). Following transfection cells were incubated for 48 h at 37°C, 5% CO_2_. Folic acid (Thermo Fisher Scientific) was reconstituted in sterile PBS, and applied to cells at the time of transfection (100 µM). C3 Transferase (Cytoskeleton) was applied to cultured MDCK cells (2 µg/ml) for 4 h prior to fixation, whereas inhibition by Y-27632 (Millipore) and Blebbistatin (Sigma-Aldrich) was applied for 90 min at the concentrations of 20 µg/ml and 50 µg/ml, respectively. W7, ML-7, and SKI-1 (Sigma-Aldrich) were utilized at the respective concentrations of 50 µM and 25 µM and 5 µM for 3 h prior to fixation.

### Chick embryo culture and chemical inhibitor use

Stage 7/8^−^ embryos were isolated from fertile eggs with a paper ring and placed on a bed of thick albumin in a 35 mm dish flooded with M199 media. Embryos were incubated for 90 min at 37°C in with distinct combinations of Folic acid (100 µM), Y27632 (20 µg/ml), and blebbistatin (50 µg/ml). Following this time period, the embryos were isolated from the paper rings, laid flat on glass slides and fixed with either 4% PFA or 2% TCA.

### Immunofluorescent labeling of MDCK cells, chick embryos, and cryosections

MDCK cells were rinsed with PBS (15 min) and fixed with 4% Paraformaldehyde (Thermo Fisher Scientific), followed by permeabilization with PBS containing 0.05% Triton-X (Thermo Fisher Scientific) for 10 min (for detection of phosphorylated epitopes). For staining of non-phosphorylated proteins, cells were fixed for 10 min in methanol. Whole chick embryos or glass slide mounted cryosections (10 µm) were fixed in 4% PFA or 2% TCA for staining of phosphorylated molecules, incubated in a solution of 100 mM Tris, pH9 for 15 min in a pressure cooker and rinsed in 0.1% PBS-T. Primary antibody incubations were performed at 4°C with gentle agitation for 12–16 h in either 4% Bovine Serum Albumin, BSA (Sigma-Aldrich) in 0.1% PBS-Tween (PBS-T, Tween, Thermo Fisher Scientific) for detection of phosphorylated epitopes, or in 4% non-fat dry milk, NFDM in 0.1% PBS-T for non-phosphorylated epitopes. The following primary antibodies were used at the indicated concentrations: rabbit α-Shroom3, 1:1000 dilution (Muccioli et al., 2016); α-Flag (F1804, Sigma-Aldrich), mouse α-V5 (Invitrogen, 37-7500), 1:500; mouse α-β-catenin (BD Biosciences, B610153), 1:500; rabbit α-β-catenin (sc-7199, Santa Cruz Biotechnology), 1:500; rabbit α-ZO-1 (61-7300, Invitrogen), 1:500; mouse α-Ap2α (3B5-c, DSHB) 1:1000; rabbit α-Folr1 (AB67422, Abcam), 1:50; non-muscle myosin II SerP20 light chain (PA1-26470, Thermo Fisher Scientific), 1:200; and myosin light chain kinase TyrP464 (sc17182-R, Santa Cruz Biotechnology), 1:50. Next, samples were rinsed in three 10-min washes with 0.1% PBS-T, then a 1–4 h incubation with one or more of the following secondary antibodies (Life Technologies; A11001, A11008, A21203, A21207) at a 1:1000 dilution in 0.1% PBS-T, 4% BSA or NFDM. Transwell membranes were then washed in 0.1% PBS-T, removed from the plastic insert with a scalpel and mounted on glass slides with Fluorogel (Electron Microscopy Sciences).

### Forl1 mouse allele generation and analysis

Mice harboring a floxed allele of the *Folr1* gene were generated in the lab of RHF, by generating a target construct that included floxed sites flanking exon 5 and 6 (Fig. S5A), which is designed to be excised upon Cre- protein expression leading to a frame-shift and an earlier termination codon. ES-positive clones harboring the targeted allele were generated and screened with standard methods (Bouabe and Okkenhaug, 2013). The flox and wild-type Folr1 alleles were detected with the following primers: forward (5′ CCAGCCAGGGTTACCTAGTG 3′) and reverse (5′ CAGGGTTTGGTTTCAAGCAC 3′), detecting a 657 base pair wild-type *Folr1* allele, and an 850 base pair flox allele (Fig. S5B). To ensure that cre action results in genetic ablation and loss of protein expression, the *Folr1^flox^* line was crossed with the *le-cre* driver line (Ashery-Padan et al., 2000) to generate *Folr1^flox/flox^; le-cre* embryos. Cre-positive lenses were dissected and analyzed for DNA recombination using the following primers: forward (5′ CTTTCTAGAGAACTTCCTCGACGGTATCGATAAGC 3′) and reverse (5′ CCCACAGGACACTCGTTATGCCC 3′), to detect a 270 bp recombination band (Fig. S5C). E18.5 cryosections were also immunofluorescently labeled with a Folr1 antibody to demonstrate loss of protein expression (Fig. S5D–G).

### Quantitative analyses and statistical methods

For apical constriction analysis of MDCK cells, the junctional outline of transgenic cells at an apical and basal plane were traced using Zen (Zeiss) software tools. The apical/basal area ratios (ABAR) was calculated and their means determined (Excel). The means, standard deviation, and number of cells analyzed are found in Tables 1 and 2. Pivot tables were used in excel to generate the binned data graphs (Excel). Because it was determined that some of the experimental groups were not normally distributed (Shapiro–Wilk), the non-parametric Mann–Whitney U-test was performed to identify experimental groups with significant differences. Chick neural epithelial cell area was quantified using the junctional signal from antibody labeling from six images within the neural epithelium of 6–10 embryos from each experimental group. Each of the six images was overlaid with a 3×3 grid and a random number generator was used to select 3, 50 µm^2^ regions from the right and left of the midline. The apical area of all cells within each selected region were measured (Zen, Zeiss).

Chick neural epithelial and surface ectoderm fluorescent intensities of pMLC was measured by tracing 100–200 randomly orientated bi-cellular junctions from at least three regions of 2–3 embryos of each experimental group using FIJI software (https://imagej.net/Fiji). Mean neural epithelial cell junctional intensity values were normalized by calculating the ratio of neural epithelial/surface ectoderm signal. pMLCK junctional intensity of wholemounted embryos was similarly calculated but instead using non-junctional signal as a normalization factor. pMLCK quantification in chick sections was calculated by measuring the normalized signal intensity along a 50 µm line placed ∼3µm beyond the apical membrane. Eight lines were measured from sections of 3–4 embryos in each experimental group and the mean was calculated across every position at ∼0.16 µm intervals along the line. Mouse embryo neural epithelial pMLC and pMLCK was calculated by measuring and calculating the normalized intensity of the apical signal along the apical junctional complex from sections of 4–7 embryos of each genotype. The Mann–Whitney U-test was performed to identify experimental groups with significant differences with the exception of Fig. 5F which used Student's *t*-test.

Paper showing reduced pMLC after MLCK depletion Myosin light chain kinase regulates hearing in mice by influencing the F-actin cytoskeleton of outer hair cells and cochleae.

## Supplementary Material

Supplementary information
