# Supplementary material for: Folic acid modifies the shape of epithelial cells during morphogenesis via a Folr1 and MLCK dependent mechanism
Source: Biol Open. 2019 Jan 15;8(1):bio041160. doi: 10.1242/bio.041160 (PMC6361208; doi:10.1242/bio.041160)
Supplement: Supplementary information [file biolopen-8-041160-s1.pdf]

S1

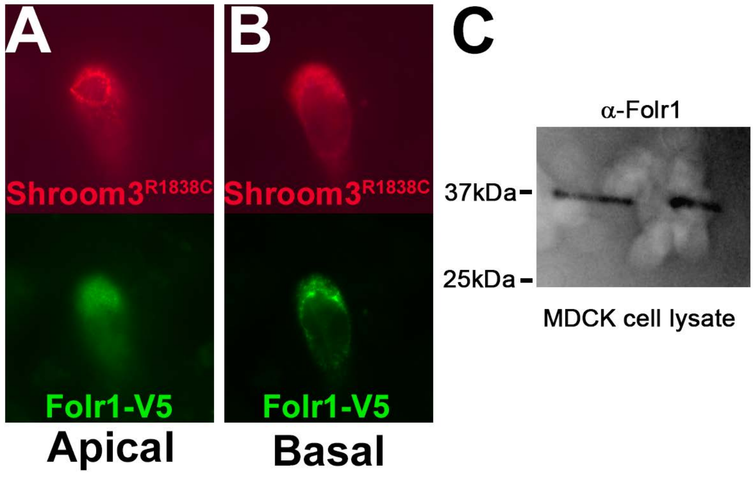

Figure S1. Shroom3 and Folr1 protein co-expression. (A-B) An apical and basal view of a representative MDCK cell expressing both the Shroom3^{R1838C} and Folr1 expression vectors. Note that apical constriction occurs in cells expressing both proteins. (C) MDCK protein lysates were isolated and immunoblotted with a folr1 antibody. The numbers to the left indicate the approximate size of the protein detected which is consistent with its expected size.

Biology Open • Supplementary information

S2

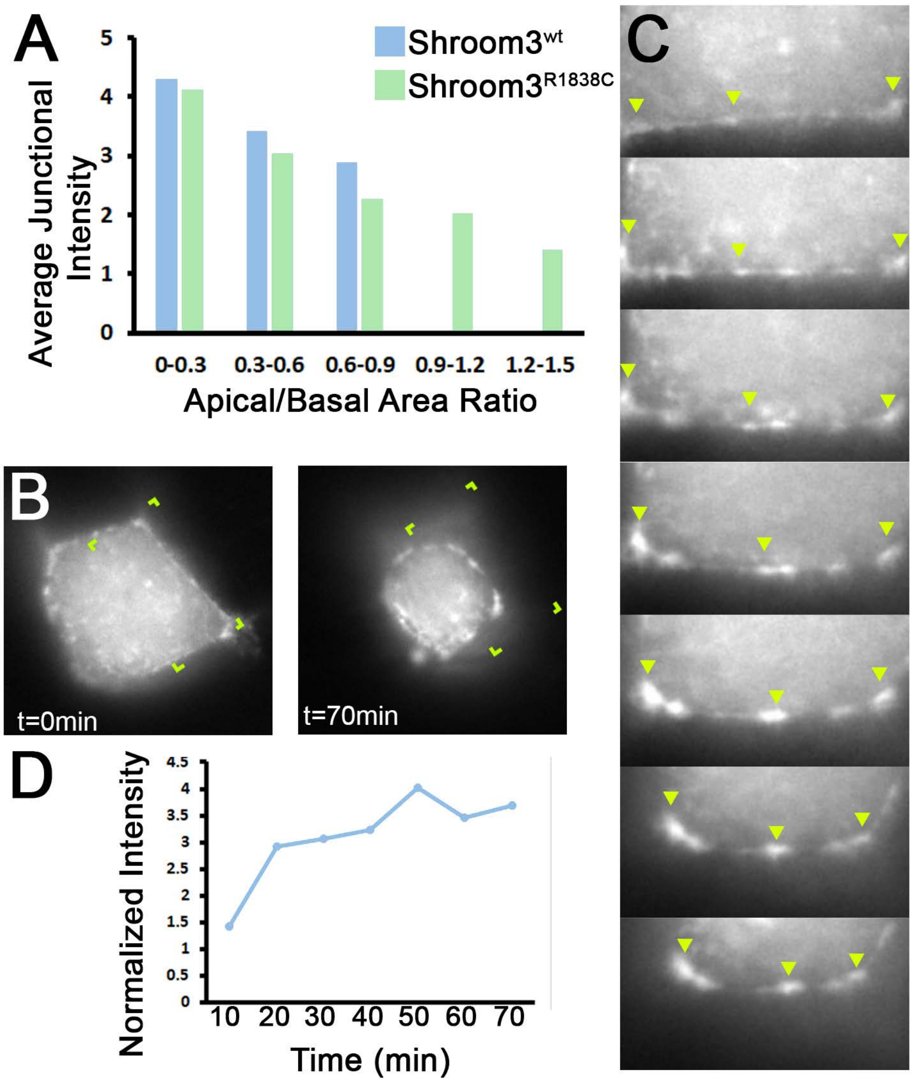

Figure S2. Transgenic Shroom3 junctional intensity increases during apical constriction. (A) The apical junctional intensity of MDCK cells transfected with Shroom3^{wt} or Shroom3^{R1838C} was measured simultaneously with the corresponding cell's apical area following immunofluorescent labeling with a Shroom3 antibody. The average junctional Shroom3 immunofluorescent intensity of cells were calculated from binned groups possessing apical/basal area ratios (ABAR) of increments of 0.3. Note that as the ABAR ratio decreased, the junctional intensity increased. (B) Still frames of a Shroom3^{wt}-mcherry transgenic MDCK cell imaged before and after apical constriction. The yellow brackets indicate the magnified image in panel C. (C) Still frames from a time-lapse video of a Shroom3^{wt}-mcherry transgenic MDCK undergoing AC with a magnified view of an apical junction. The arrowheads represent spots of intense Shroom3 protein that move closer together with time. (D) The normalized intensity of the junction observed in C is plotted with time. Note that the increase in fluorescent intensity with time suggests that the density of Shroom3 protein may increase.

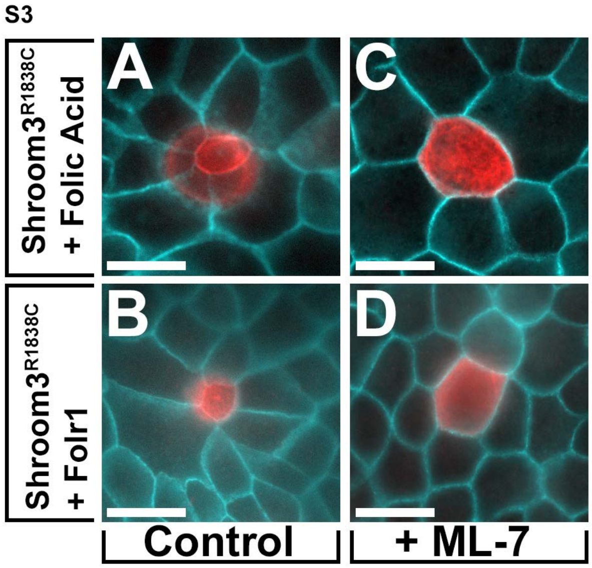

Figure S3. ML-7 inhibits folic acid and folr1-mediated rescue of Shroom3 function. (A-D). Apical views of representative MDCK cells expressing Shroom3^{R1838C} treated with or without exogenous folic acid or co-expressed with folr1. Shroom3 (red) and β-catenin (turquoise) are immunofluorescently labeled. Note that ML-7 prevents apical constriction in Shroom3-folic acid and Shroom3-Folr1 positive cells.

Biology Open • Supplementary information

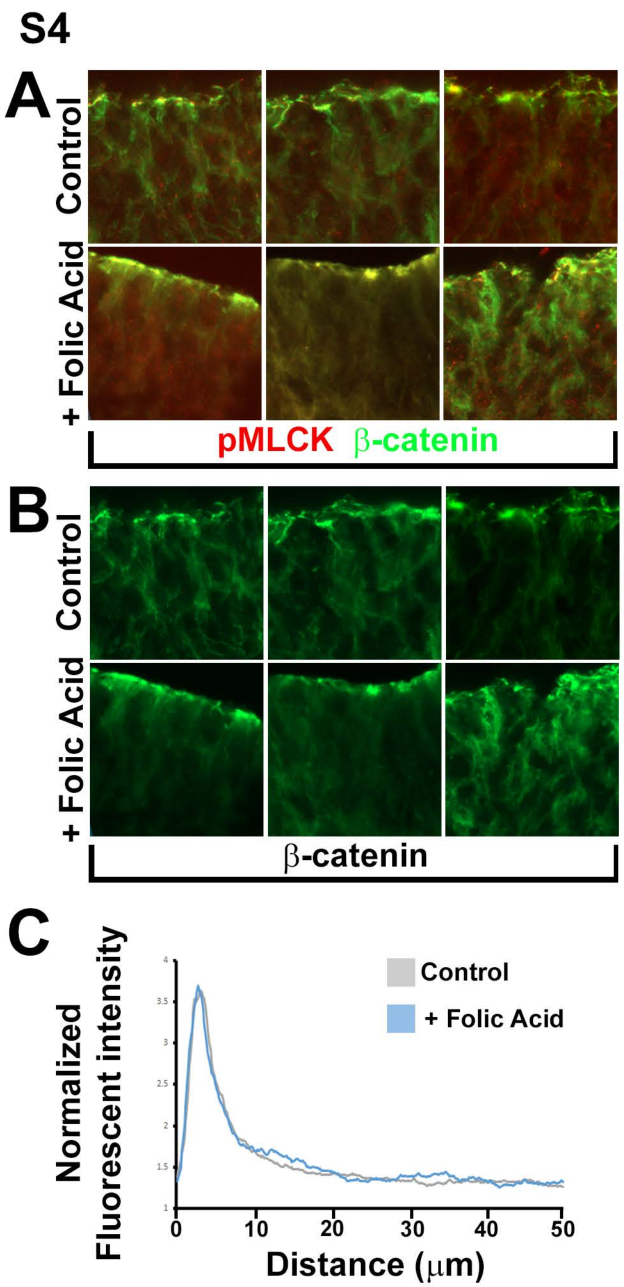

Figure S4. Confirmation that beta-catenin fluorescent intensity is unaffected by exogenous folic acid. (A-B). Co-immunofluorescent labeling of pMLCK and β-catenin (A) or only β -catenin labeling (B) from the images in figure 5D-E. (C) The fluorescent intensity of β-catenin along a 50 μm apical/basal line was measured from the same images of cryosections used in (5D-E) and the mean intensity along the line was calculated. Note that unlike pMLCK, a difference between the experimental groups was not observed.

Biology Open • Supplementary information

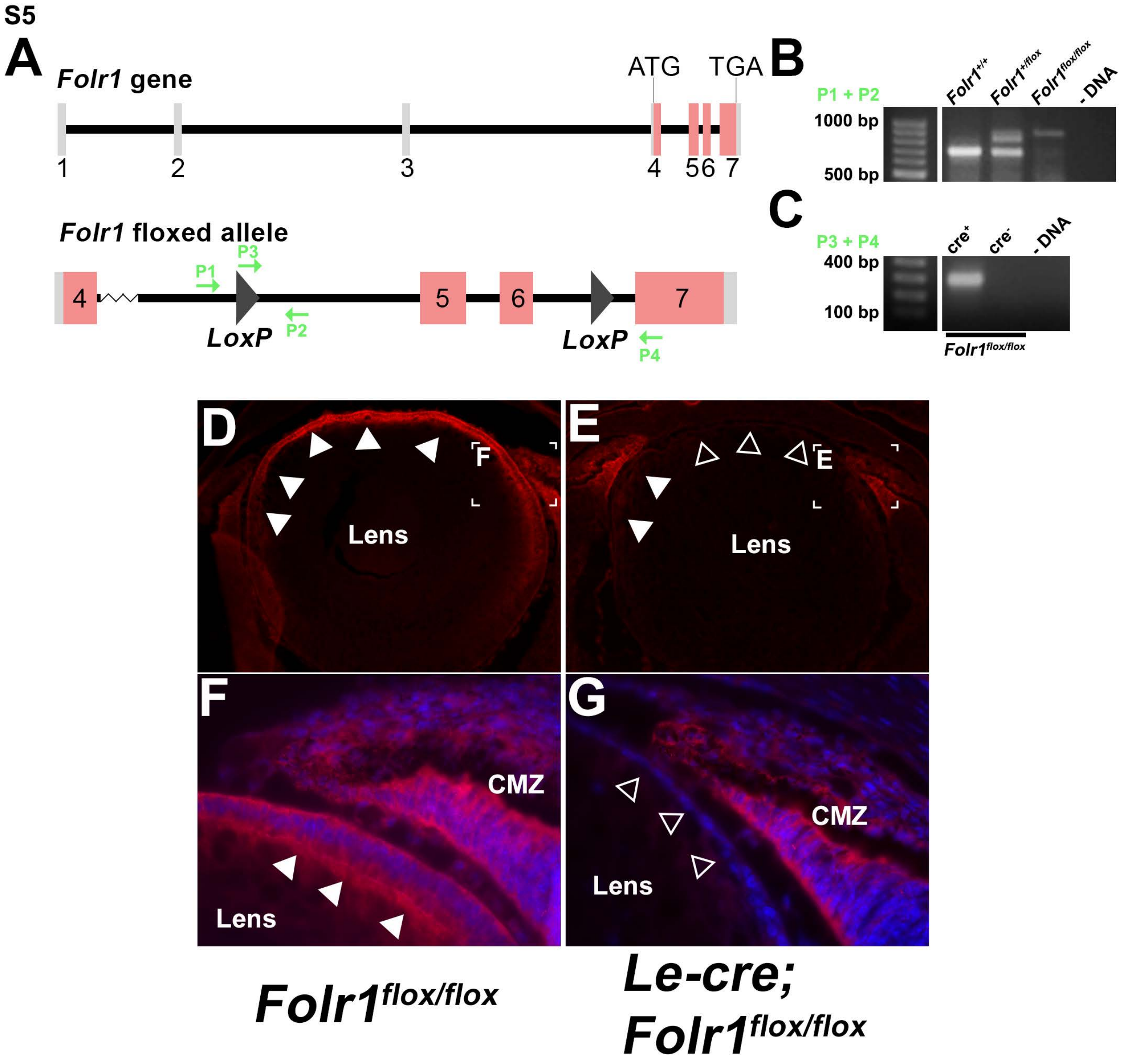

Figure S5. Confirmation of Folr1 recombination and protein loss following conditional deletion. (A) Diagram of the exons and location of the start and stop sites of the endogenous mouse Folr1 gene (top). The location of the LoxP sites between exons 4 and 7 are diagrammed along with the location of the PCR primers used for genotyping and recombination detection. (B) Genotyping PCR results generated from tail samples of pups born from a *Folr1*$^{+/flox}$ x *Folr1*$^{+/flox}$ cross. (C) PCR results on DNA isolated from lenses dissected from embryonic mouse eyes (E17.5) that are either *Folr1*$^{flox/flox}$*; le-cre* or *Forl1*$^{flox/flox}$. Note the presence of the recombination band indicating that the region between the LoxP sites has been deleted. (D-G) Histological cryosections of E18.5 mouse embryos with the indicated genotype immunofluorescently labeled with a Folr1 antibody (red) and Hoechst (blue). Closed arrowheads indicate positive signal while open arrowheads indicate the absence of signal. Note that Forl1 protein is specifically removed from the lens but not the ciliary margin (CMZ) upon le-cre mediated recombination.

Biology Open • Supplementary information

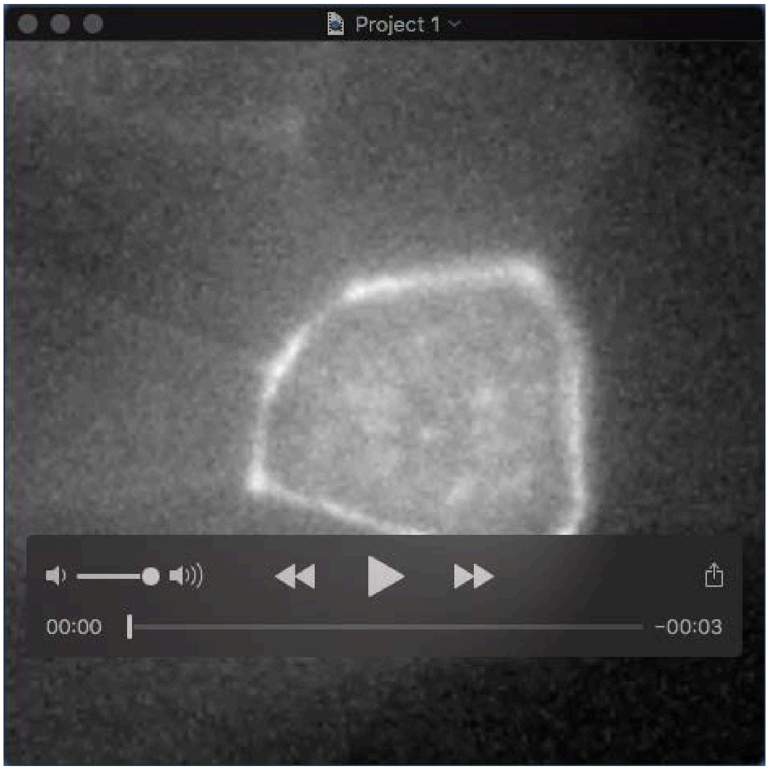

Movie 1. Folic acid permits apical constriction in Shroom3^{R1838C} positive cells. Shroom3^{R1838C}-mcherry positive MDCK cell treated with exogenous folic acid. Each frame represents 10minutes. Note that exogenous folic acid was added at the movie's onset.
